# Limb Repair Using the Extended Chimeric Fasciocutaneous Scapular and Latissimus Dorsi Muscle (ECS-LD) Flap: A Case Series

**DOI:** 10.7759/cureus.90467

**Published:** 2025-08-19

**Authors:** Sarah E Moffitt, Madison M Patrick, Claudia Cruz, Kristen Whalen, Bilal Koussayer, D’Arcy Wainwright, Nicole K Le, Jared Troy

**Affiliations:** 1 Department of Plastic Surgery, University of South Florida, Tampa, USA; 2 Department of Surgery, Florida State University College of Medicine, Tallahassee, USA

**Keywords:** chimeric flap, extremity soft tissue defect, flap reconstruction, latissimus dorsi flap, microsurgery

## Abstract

We present four reconstructive cases of large soft tissue extremity defects utilizing a modification of the classic scapular-latissimus dorsi chimeric flap, which we refer to as the extended chimeric scapular and latissimus dorsi muscle flap (ECS-LDF). A 57-year-old male presented with a right lower extremity Gustilo IIIB fracture and an open knee joint with associated soft tissue loss following a motorcycle accident. An ECS-LDF (397 cm²), based on the subscapular artery, was harvested for coverage. The scapular skin paddle was inset over the knee, while the latissimus dorsi muscle was placed inferiorly over the leg defect and covered with a split-thickness skin graft (STSG). A five-year-old girl sustained multiple left lower extremity fractures and circumferential degloving of the knee due to a crush injury sustained in an auto-pedestrian trauma. The scapular portion (144 cm²) of the ECS-LDF was used to resurface the popliteal fossa, while the latissimus dorsi muscle and overlying STSG (351 cm²) covered the anterior knee and lateral leg. A 61-year-old male presented with a Gustilo IIIB fracture with extensive, near-circumferential degloving and soft tissue loss of the lower third of the left lower extremity. The latissimus dorsi muscle and scapular skin paddle of the ECS-LDF (310 cm²) provided complete soft tissue coverage. A 30-year-old female sustained a traumatic right upper extremity injury involving avulsion of the extensor tendons, median and ulnar nerves, and open radius and ulna fractures with overlying soft tissue loss. She underwent an ECS-LDF (250 cm²) with vascularized scapular bone for composite bony and soft tissue reconstruction.

This series demonstrates the unique flexibility of the ECS-LDF in the reconstruction of complex defects due to its large skin paddle and freedom to independently orient the skin and muscle flaps. Furthermore, it results in a single donor with primary closure and a single microsurgical anastomosis. We believe the ECS-LDF is an excellent addition to the reconstructive microsurgeon's armamentarium.

## Introduction

Plastic surgeons are often confronted with a wide array of reconstructive challenges. Microsurgical techniques allow for more sophisticated flap designs that can be tailored to the unique requirements of the patient. A common and difficult problem encountered during limb salvage is large extremity defects with exposed anatomic structures and orthopedic hardware. These patients often require coverage in an expedited fashion, can have limited vascular targets for free tissue transfer, and often require surface area coverage greater than that easily obtained by a single flap [1-3]. While multiple flaps with additional anastomosis are always an option, use of chimeric flaps that provide large, composite soft tissue components with a single anastomosis presents a better choice in this difficult patient population [3]. 

Secondary considerations include donor site morbidity, flap bulk, and ultimate contour, which can necessitate additional revision surgery. Thin fasciocutaneous flaps and muscle flaps are advantageous due to improved long-term contour [4]. When possible, it is advantageous to obtain primary closure of the donor site to eliminate the morbidity of a skin graft [5,6].

In an attempt to meet these criteria, chimeric flaps have grown in use. Here, we present four cases of large soft tissue extremity defects reconstructed with a modification to the classic scapular-latissimus dorsi chimeric flap. Extension of the cutaneous paddle to include lateral chest and axillary tissue greatly increases the size of the skin paddle, allowing for harvest of both flaps through a single incision, primary closure of the donor site, and a single anastomosis. We refer to this modification as the extended chimeric scapular and latissimus dorsi muscle flap (ECS-LDF) and believe it is a valuable reconstructive option for complex extremity wounds.

## Case presentation

Methods

ECS-LDF Operative Technique

At our institution, lower extremity computed tomography angiography (CTA) is routinely performed to assess potential microsurgical targets. When designing the skin paddle for the ECS-LDF, a hand-held Doppler is utilized to locate the dominant perforator arising through the triangular space. A transverse skin paddle is designed with the scapula displaced inferiorly to maximize the flap width. The medial and lateral extent can include the tissue crossing the midline of the back and up to the anterior axillary line.

**Figure 1:**
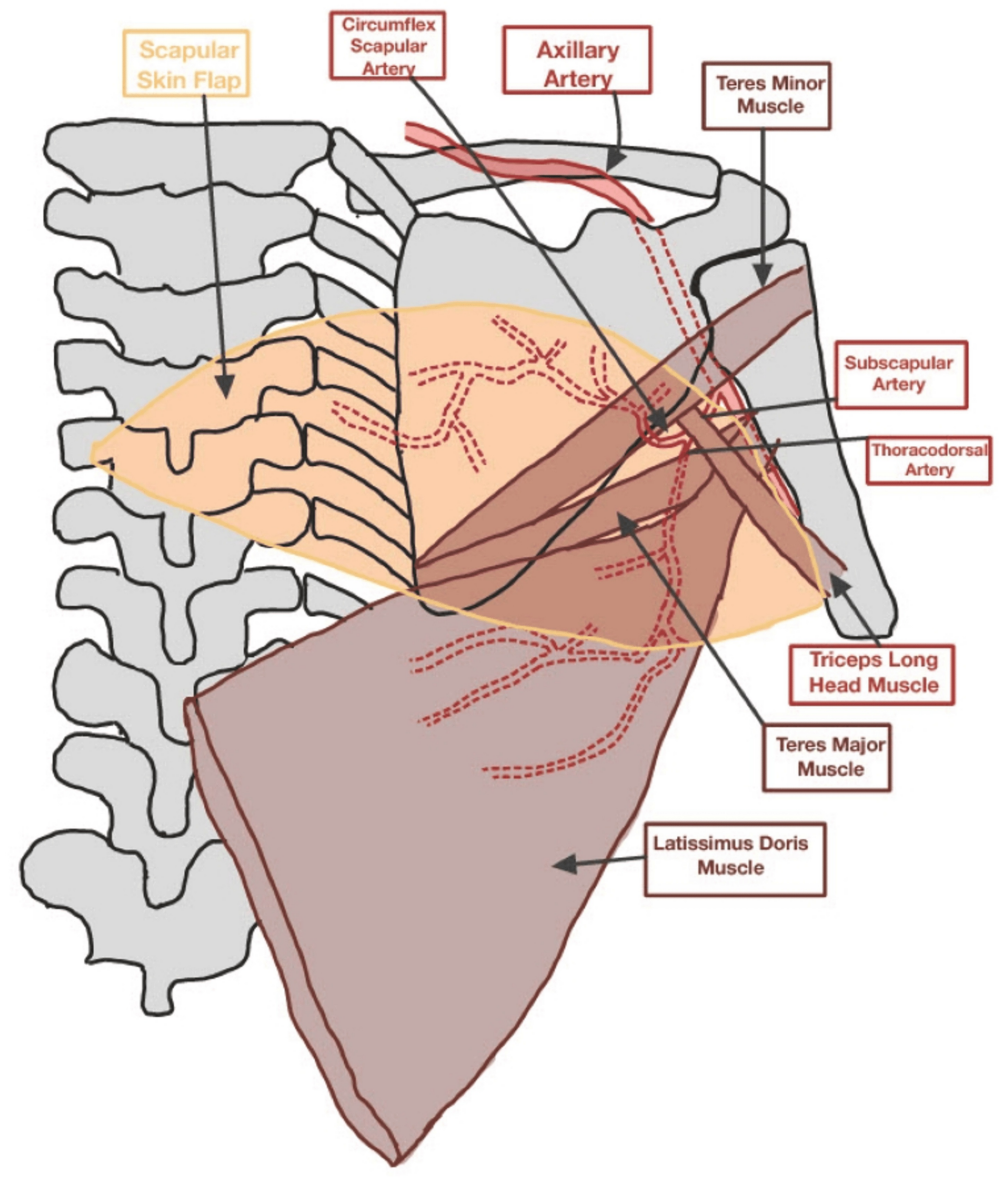
Schematic of the extended chimeric scapular and latissimus dorsi muscle flap (ECS-LDF). This skin paddle is marked in the transverse dimension to maximize flap size while still facilitating primary closure. The scapular fasciocutaneous flap is based on the circumflex scapular vessels, and the latissimus dorsi muscle flap is based on the thoracodorsal vessels; both sets of vessels originate from the subscapular artery and vein. This image is created by the author, Bilal Koussayer, of this study.

The flap is then raised in the standard fashion, with the circumflex scapular pedicle dissected back to the thoracodorsal vessels through the triangular space. The latissimus dorsi muscle is then raised through the same incision. The thoracodorsal vessels are dissected back to their origin (Figure 1). Perfusion to the two flaps is evaluated with indocyanine green angiography. Once appropriate perfusion is confirmed, the scapular artery and vein are ligated, and the flap is transferred to the recipient site. Drains are placed in the donor site and closed primarily.

Case 1

**Figure 2:**
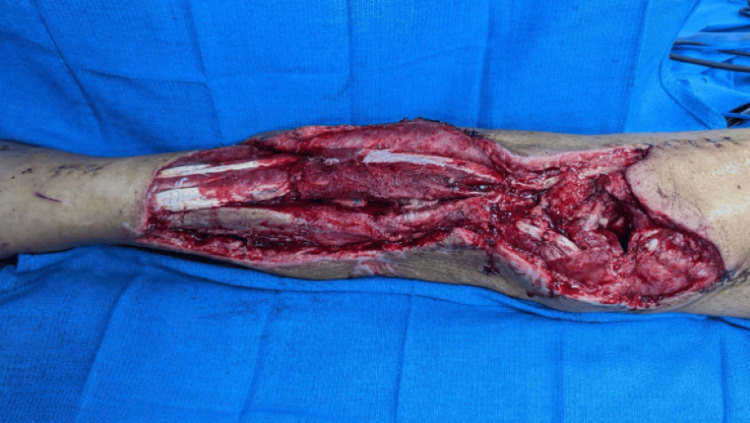
Pre-operative photograph of Case 1 depicting a full-thickness wound of the right lower extremity measuring 397 cm², with exposed bone, muscle, fascia, and tendon.

A 57-year-old male presented following a motorcycle accident with a right lower extremity (RLE) Gustilo IIIB injury and traumatic knee arthrotomy. The patient underwent debridement and open reduction and internal fixation (ORIF) of the tibia and fibula fractures, done by the orthopedic surgery team. The right lower extremity wound was temporized with negative pressure wound therapy (NPWT). Plastic surgery was consulted for soft tissue coverage of a full-thickness wound with exposed bone, muscle, fascia, tendon, and hardware (Figure 2).

**Figure 3:**
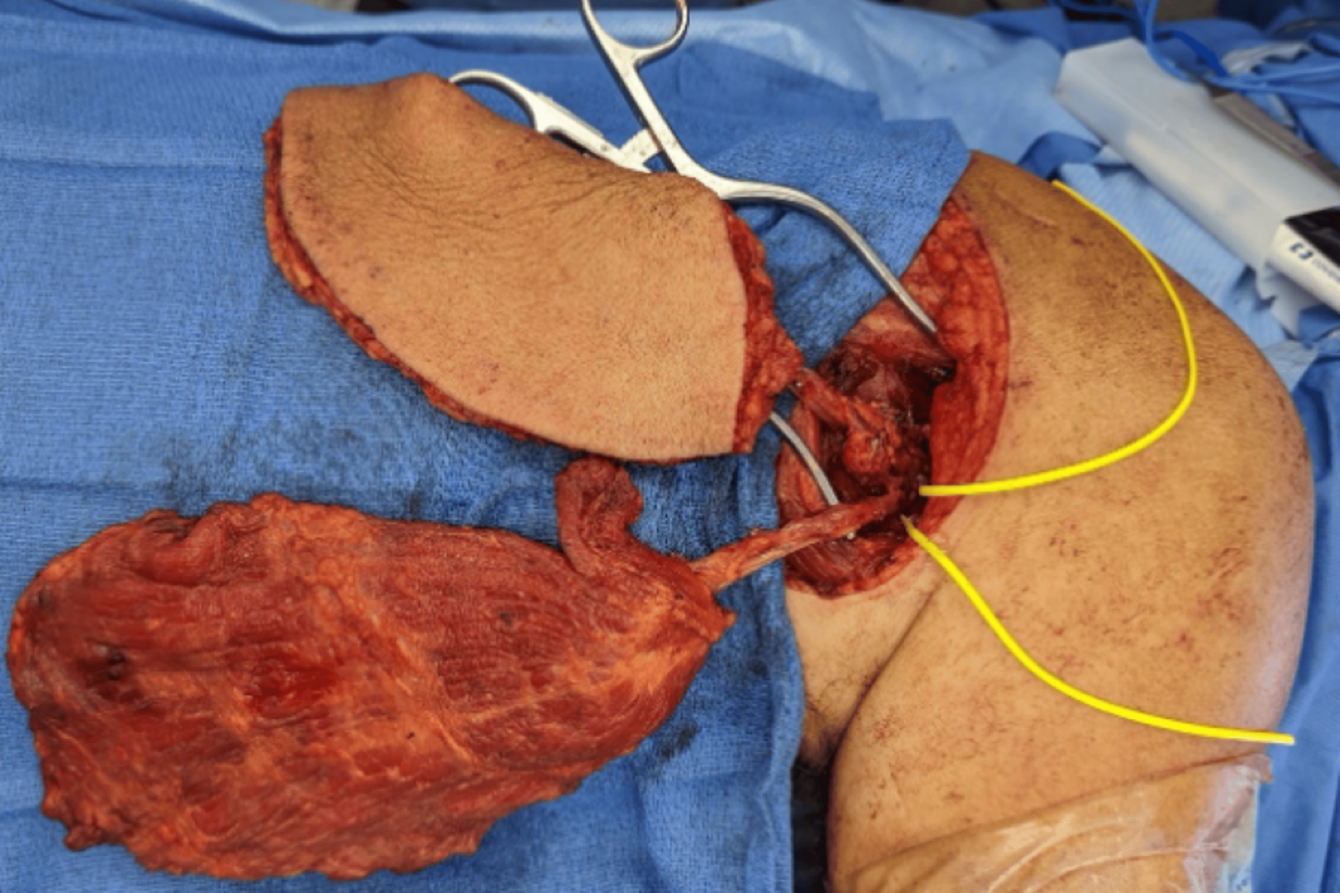
Intra-operative photograph of Case 1 depicting the ECS-LDF isolated from the subscapular artery. ECS-LDF: extended chimeric scapular and latissimus dorsi muscle flap

**Figure 4:**
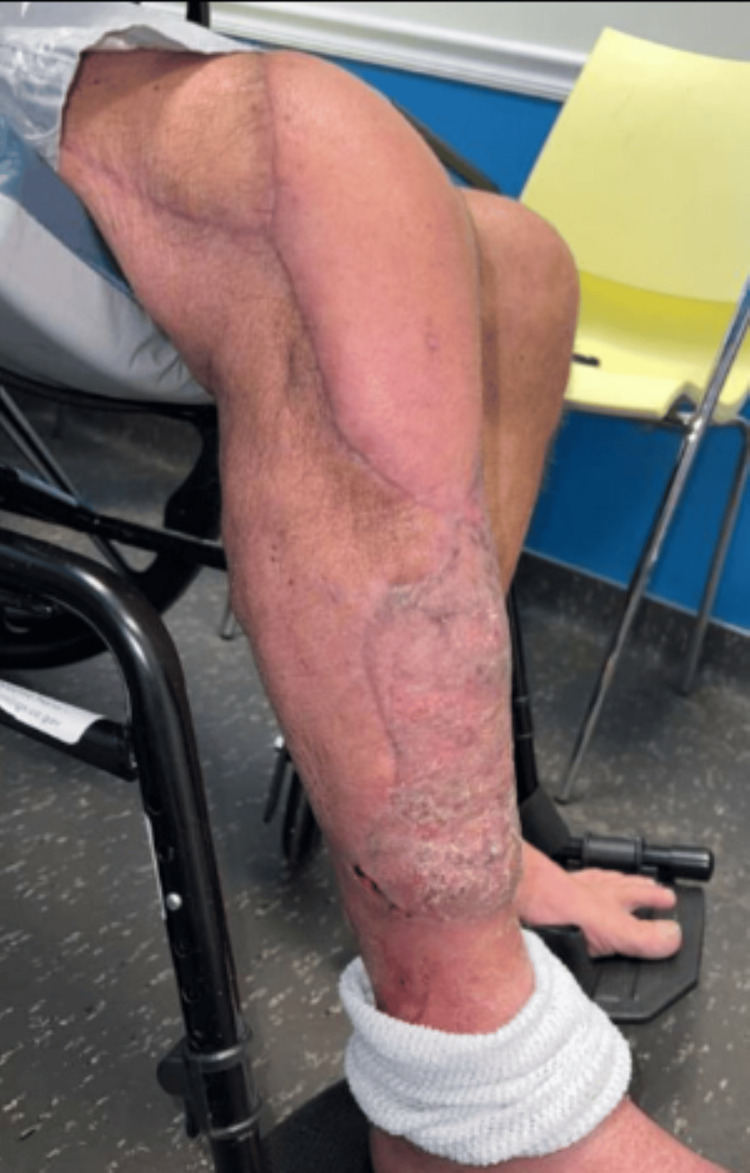
Two-month post-operative photograph of Case 1 showing complete healing following right lower extremity reconstruction with ECS-LDF. ECS-LDF: extended chimeric scapular and latissimus dorsi muscle flap

The patient underwent definitive reconstruction with an ECS-LDF (397 cm^2^) based on the subscapular artery (Figure 3). The scapular skin paddle was inset over the knee, and the latissimus dorsi muscle was placed inferiorly over the leg defect with an overlying split-thickness skin graft (STSG). The donor site was closed primarily. Two months post-operatively, the patient had complete wound healing at both the donor and graft sites without complications (Figure 4).

At six months post-operatively, he was weight-bearing as tolerated and continued to use a wheelchair, but was able to ambulate with assistance. Patient's post-operative return to normal functioning was complicated by difficulty acquiring physical therapy due to insurance coverage. Patient exhibited 4/5 strength of knee flexion and extension on his right side. His active range of motion of his right knee was 20-95 degrees, and passively 10-100 degrees. The patient was provided with a physical therapy referral, but unfortunately lost to follow-up after the six-month post-operative visit and has since passed away.

Case 2

**Figure 5:**
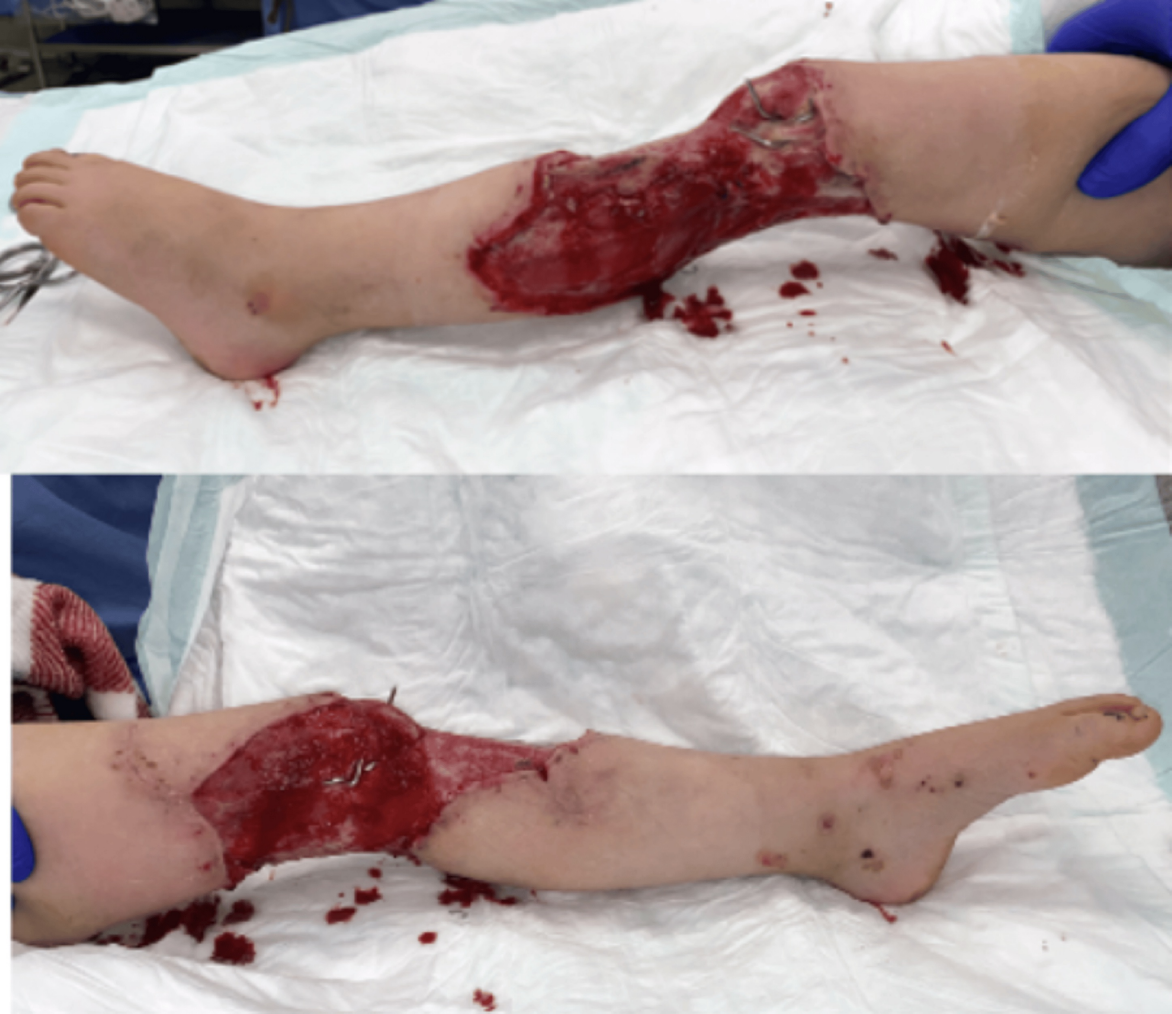
Pre-operative photograph of Case 2, showing a left lower extremity full-thickness defect with exposed muscle, fascia, bone, and hardware, measuring 324 cm².

A five-year-old female presented with a crush injury from an auto-pedestrian accident with multiple injuries, including a left distal femur and proximal tibial fracture with soft tissue degloving at the fracture sites. She underwent debridement, open reduction, and pin fixation of the femur and tibial fractures, and wound temporization with NPWT performed by the orthopedic surgery team. Following multiple debridements, she had a full-thickness defect with exposed muscle, fascia, bone, and hardware (Figure 5).

**Figure 6:**
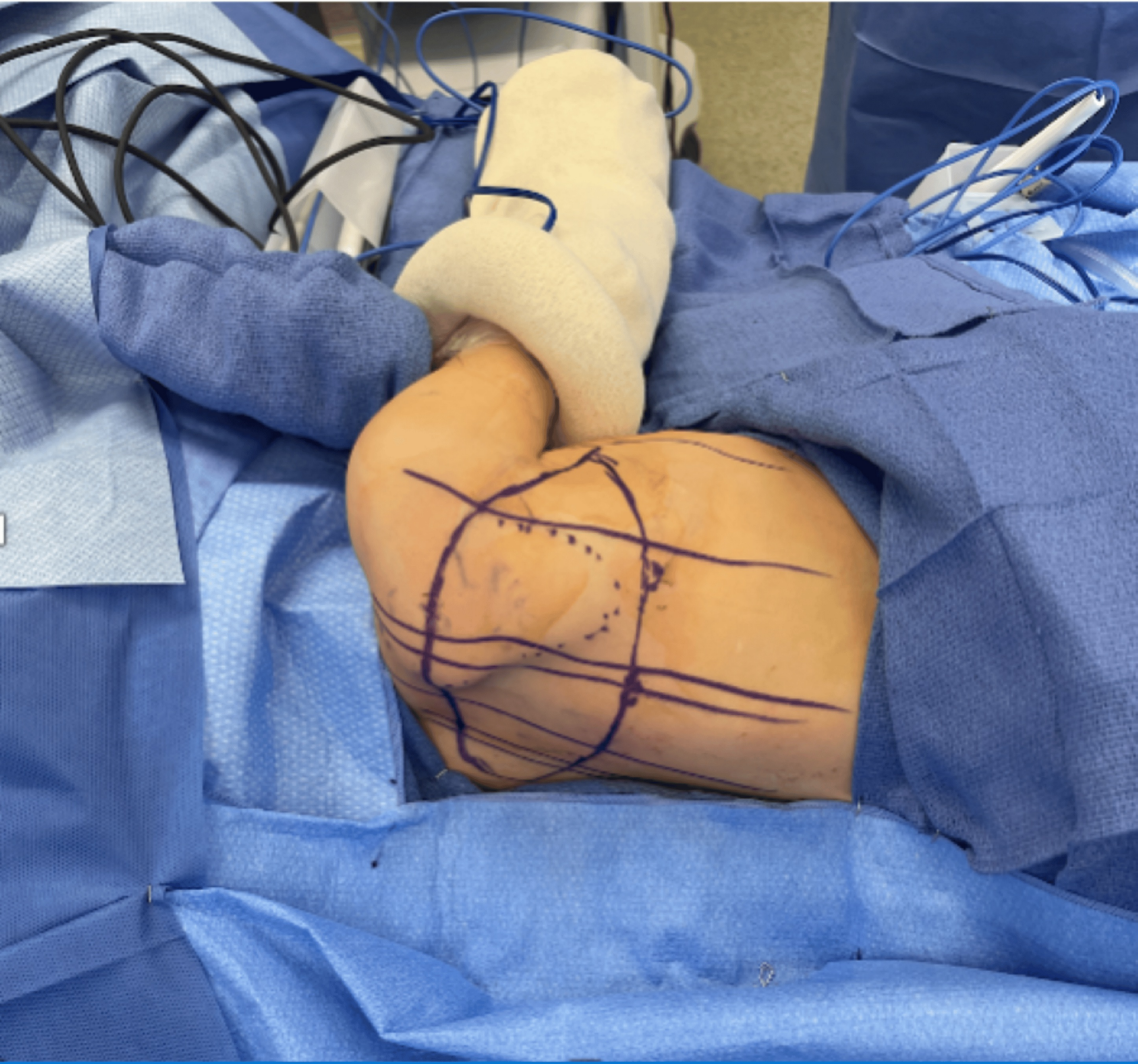
Intra-operative planning of the ECS-LDF in Case 2. The maximum width is marked by performing a pinch test with the shoulder displaced inferiorly. The medial and lateral extent of the skin paddle can extend past the back midline to the anterior axillary line. ECS-LDF: extended chimeric scapular and latissimus dorsi muscle flap

Our team performed reconstruction with a contralateral ECS-LDF (Figure 6). The scapular portion (144 cm^2^) was used to resurface the popliteal fossa, and the latissimus dorsi muscle and STSG (351 cm^2^) covered the anterior knee and lateral leg. One month post-operatively, the patient was found to have partial flap necrosis (100 cm^2^) over the distal tip. She underwent debridement and allograft reconstruction (100 cm^2^), followed by STSG (100 cm^2^), to the area of flap necrosis. Primary closure of the donor site on her right posterior thorax was achieved.

**Figure 7:**
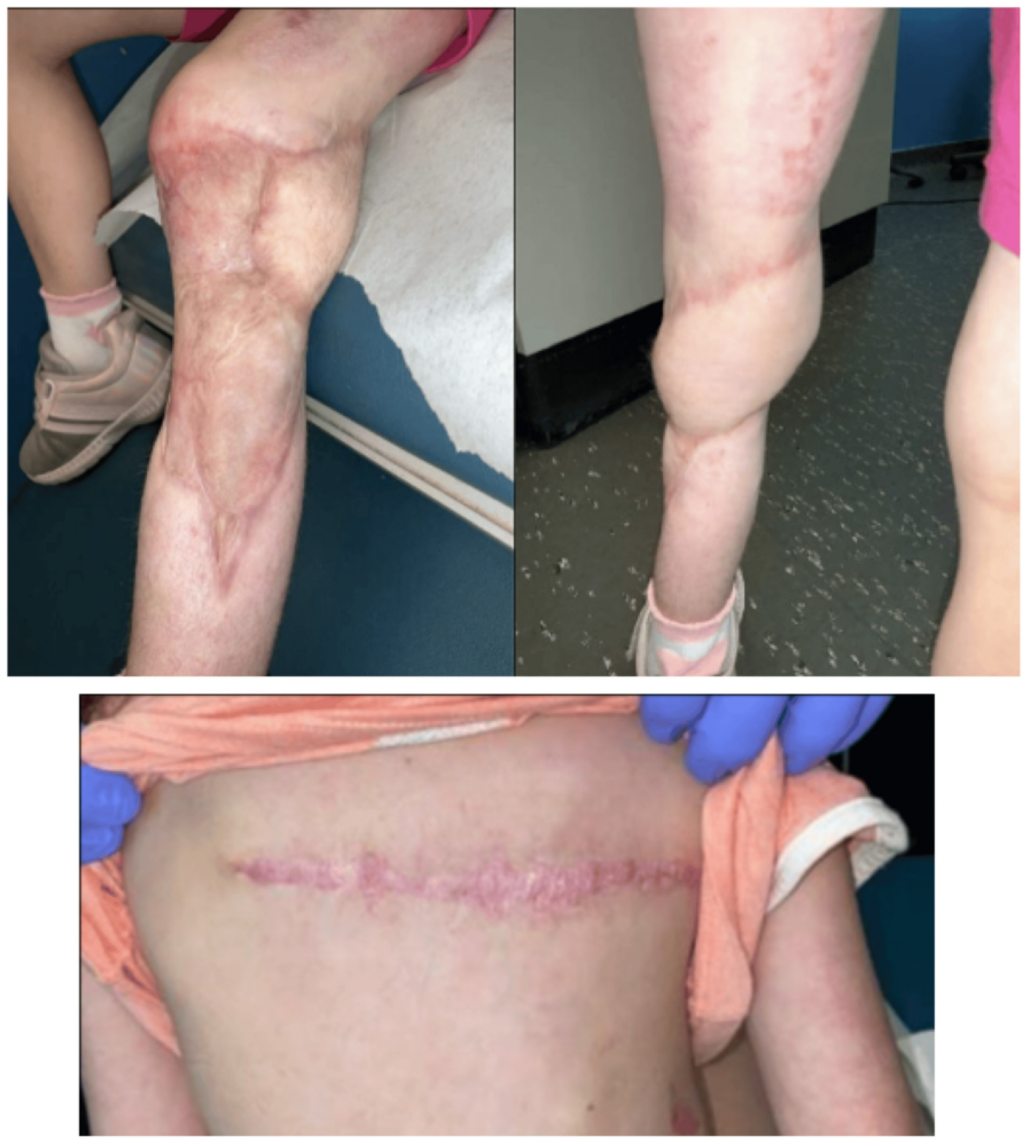
Two-month post-operative photographs of the LLE graft site and right posterior thorax donor site of Case 2. LLE: left lower extremity

**Figure 8:**
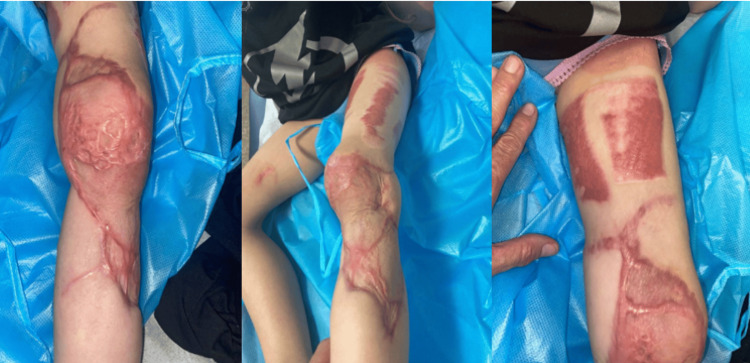
Five-month post-operative images of LLE hypertrophic scarring in Case 2. LLE: left lower extremity

She healed well and demonstrated full extension and flexion of her left knee two months post-operatively (Figure 7). At four months post-operatively, the patient exhibited foot drop of the left foot, toe walking, and decreased range of motion of the left ankle. Patient was encouraged to establish care with physical therapy, which was delayed due to insurance approval. At five months post-operatively, the patient returned to the clinic with hypertrophic scarring and scar tightness of her left lower extremity (LLE) at the skin graft donor sites and the reconstructed site (Figure 8).

**Figure 9:**
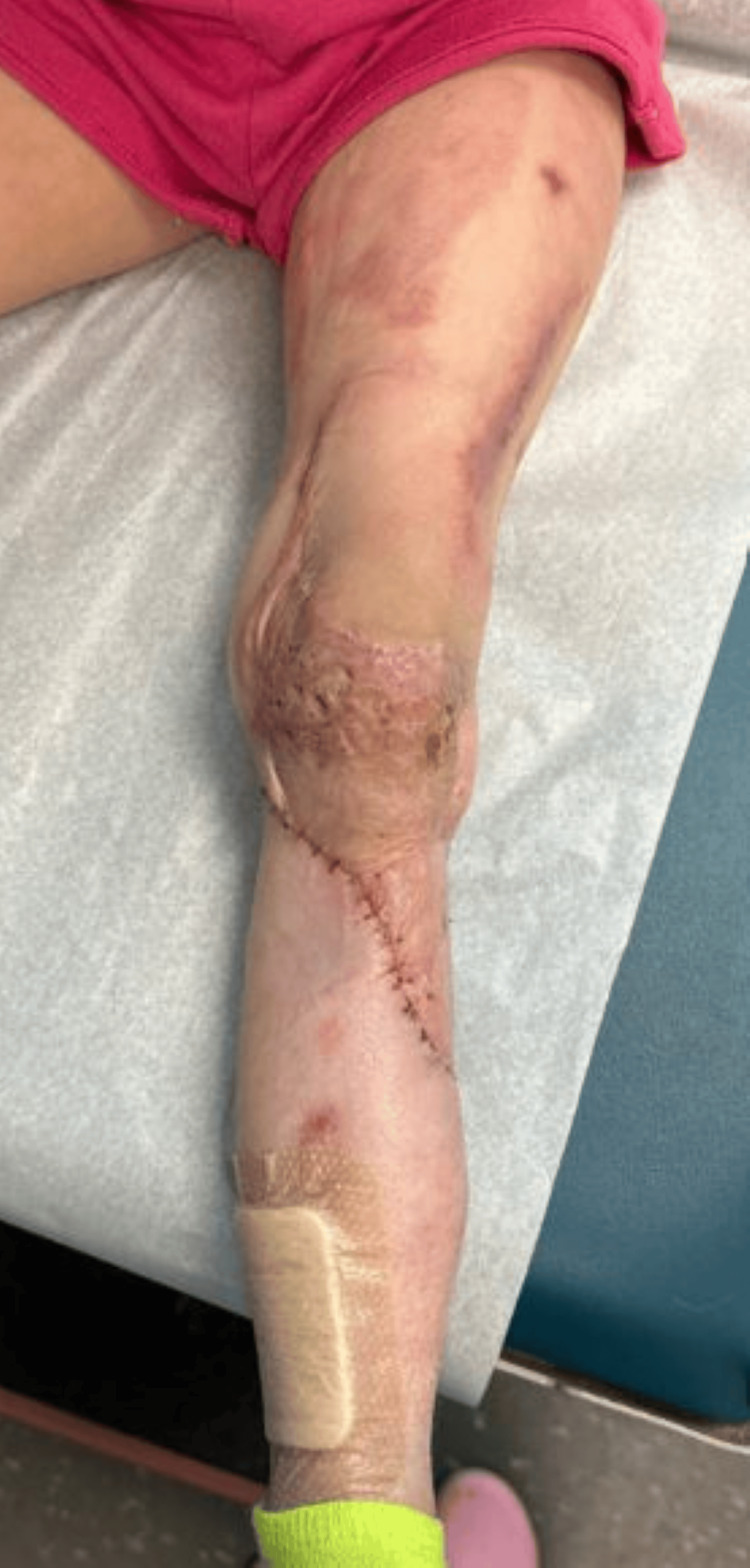
Post local tissue rearrangement of the LLE to manage tissue expansion following unsuccessful treatment with CO₂ laser therapy and steroid injections in Case 2. LLE: left lower extremity

She underwent three attempts of CO_2_ laser therapy with Kenalog steroid injections for treatment. Ultimately, the patient had tissue expansion of the LLE and subsequent local tissue rearrangement with excisional debridement for the hypertrophic scars (Figure 9). She continues to follow-up with the plastic surgery team for management of the scars.

**Figure 10:**
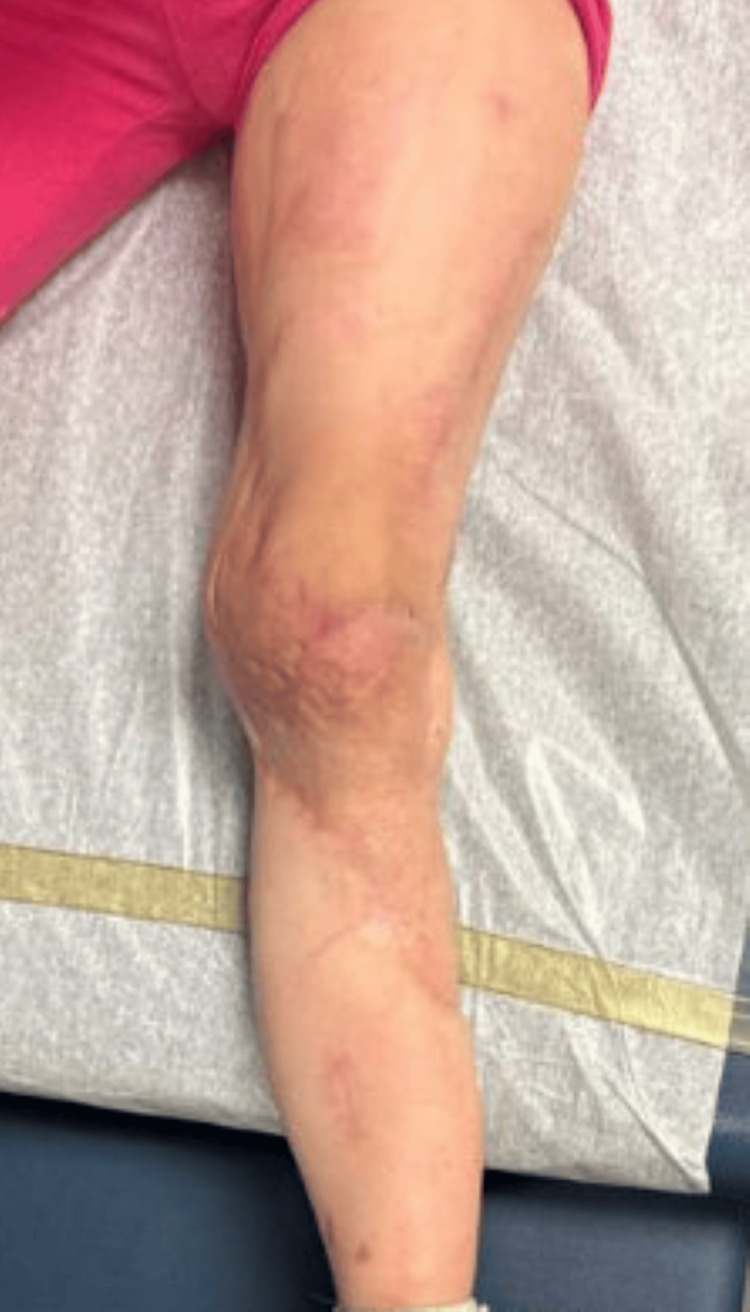
Two years post-operative follow-up with improved scarring in Case 2.

Patient continued to improve her range of motion and gait with physical therapy and was back to running and playing by one and a half years post-operatively. Patient seen most recently at two years post-operatively with greatly improved scarring of the LLE and reporting increased confidence and comfort with the appearance (Figure 10).

Case 3

A 61-year-old male was transferred to our hospital 11 days after an auto-pedestrian accident, in which he sustained a Gustilo IIIB left lower extremity fracture with near circumferential degloving. He underwent left lower extremity debridement, ORIF, and NPWT. The patient had a large soft tissue defect with exposed muscle and tibial bone. A 160 cm^2^ ECS-LDF was performed for definitive reconstruction. The latissimus dorsi muscle was inset over the exposed tibial bone, and the scapular flap was then placed over the proximal wound. A STSG was placed over the latissimus dorsi muscle and the remaining exposed muscle of the anterolateral leg. The patient healed without complication. Follow-up and case imaging are limited due to the patient being transferred back to the outside admitting hospital post-operatively.

Case 4

**Figure 11:**
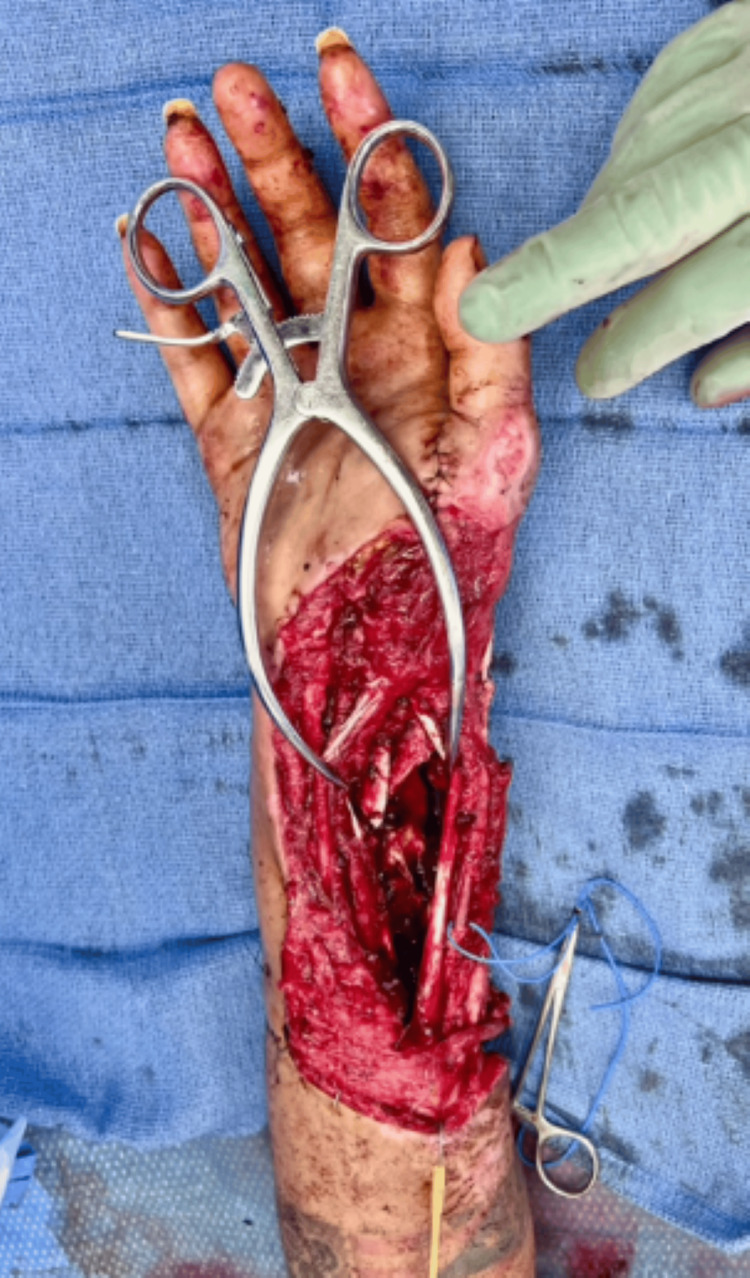
Intra-operative images of right upper extremity injury with near-circumferential soft tissue loss and exposed bone, tendons, and nerves in Case 4.

A 30-year-old female presented after a motor vehicle accident with right upper extremity open distal radius and ulnar fractures, traumatic extensor tendon avulsions, and injuries to the right median, ulnar, and radial sensory nerves (Figure 11). The orthopedic surgery team performed debridement, tendon and nerve repair, and post-operatively managed with NPWT. There was a 7 cm bony gap in the distal radius with a near circumferential soft tissue defect.

**Figure 12:**
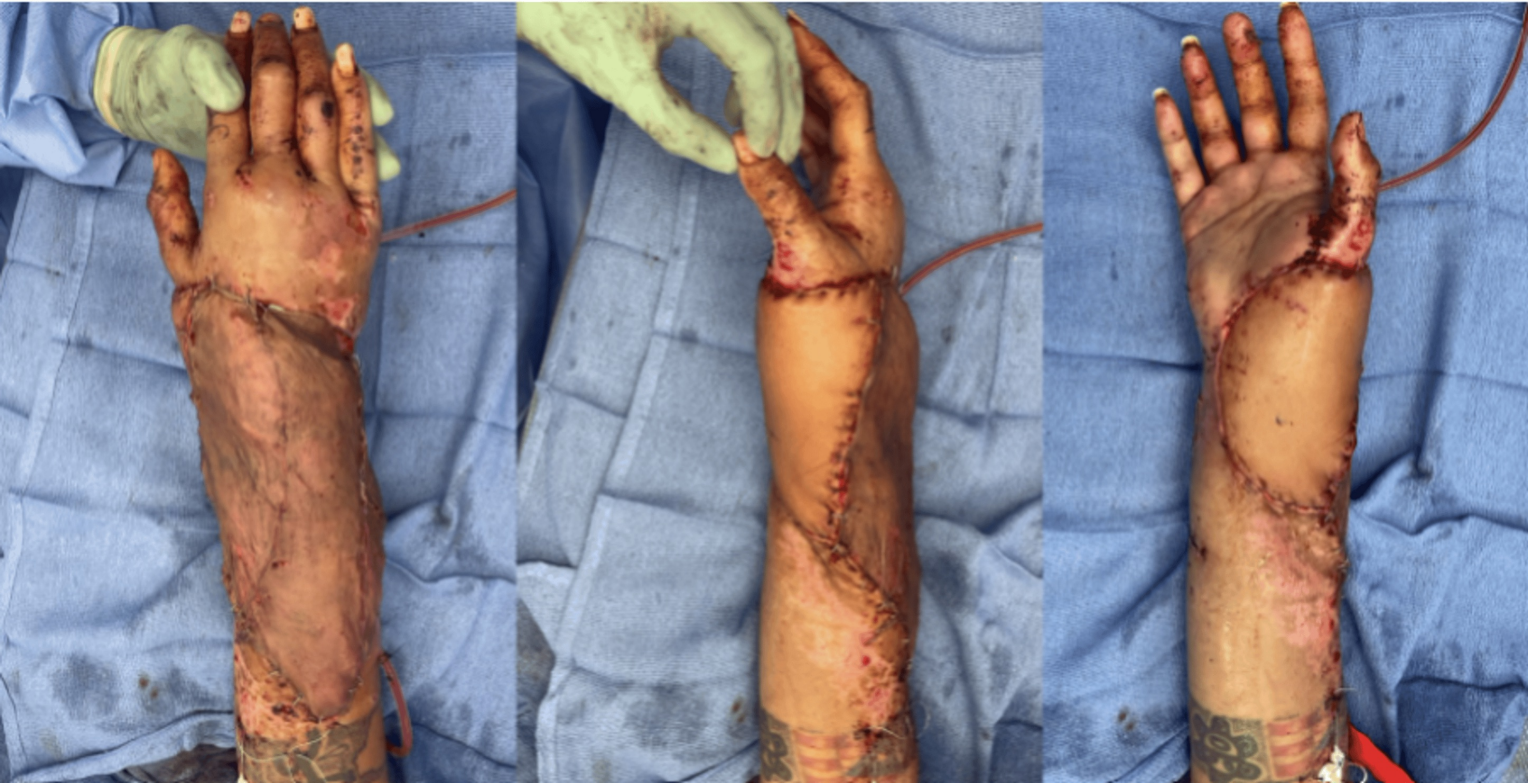
Immediate post-operative view of the ECS-LDF to the right upper extremity in Case 4. ECS-LDF: extended chimeric scapular and latissimus dorsi muscle flap

An ECS-LDF with vascularized scapular bone was harvested for composite reconstruction. The flap included a cutaneous flap measuring 240 cm^2^, a vascularized scapular bone measuring 20 cm^2^, and a muscle flap measuring 375 cm^2^. When dissecting the muscular portion of the flap, it was discovered that the patient had an anatomic variation, where the thoracodorsal and circumflex scapular arteries both directly originated from the axillary artery without a common subscapular arterial source. The independent branch of the circumflex scapular artery was ligated off the axillary artery, and a large side branch was preserved for eventual anastomosis to the thoracodorsal branch of the latissimus dorsi muscle flap. The scapular bone flap was supplied by the angular arterial branch of the thoracodorsal and the periosteal branch of the circumflex scapular arteries. The orthopedic team inserted the bone graft with rigid fixation to the radius. This was followed by anastomosis of the circumflex scapular artery to the radial artery and the thoracodorsal artery to a side branch of the circumflex scapular artery. The common venous trunk of the latissimus dorsi and chimeric scapular osteocutaneous flaps was hand-sewn to the cephalic vein, as the size difference made a coupler anastomosis unfeasible. A STSG was taken from the left upper lateral thigh and buttock for coverage of the latissimus dorsi muscle flap (Figure 12).

**Figure 13:**
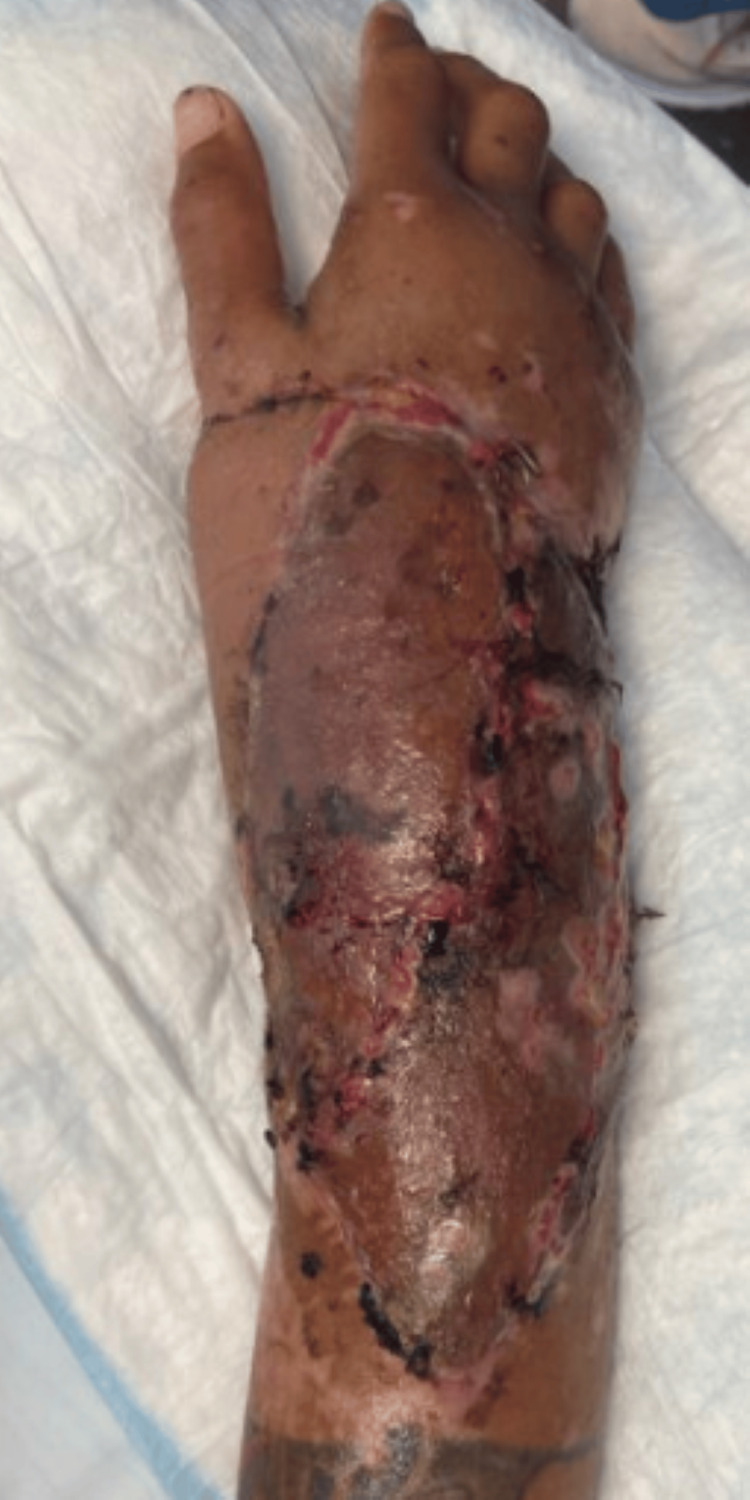
One-month post-operative image showing 90% graft take in Case 4.

**Figure 14:**
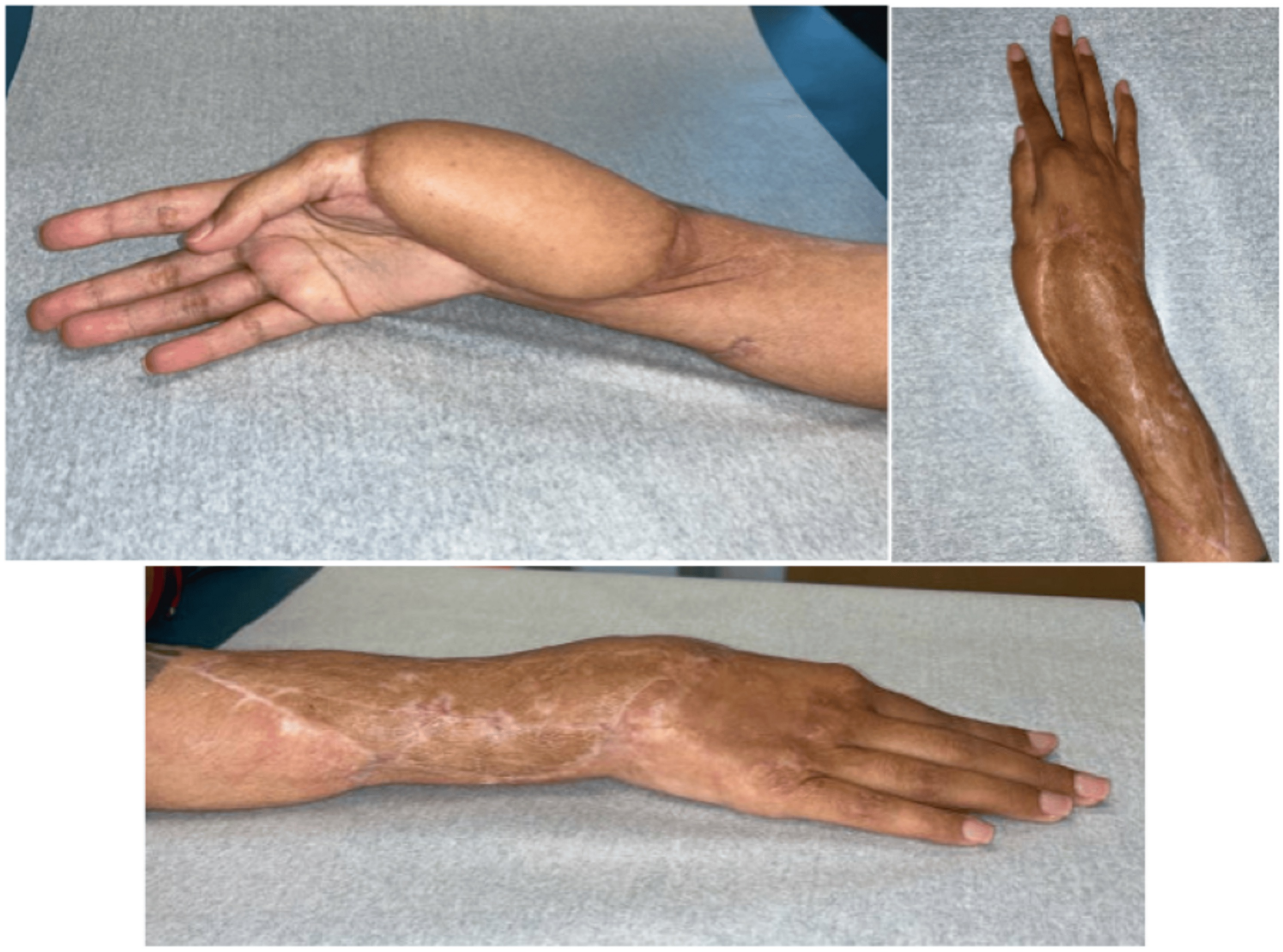
One-year post-operative images of Case 4 prior to flap debulking operation.

**Figure 15:**
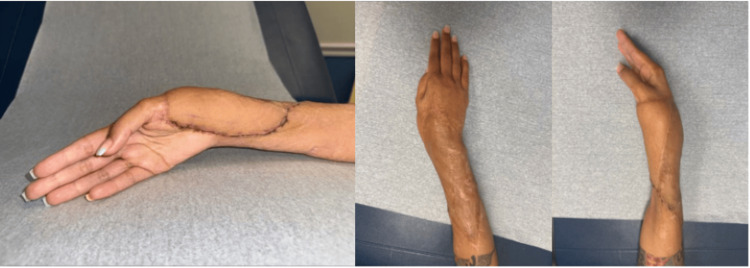
Five-day post-operative images of debulking surgery in Case 4.

The patient presented one month post-operatively with 90% graft take (Figure 13). She continued to heal appropriately and followed up with the orthopedic hand team. At three months post-operatively, the patient had a normal range of motion of her right wrist and hand. At one-year post-ECS-LDF reconstruction, she presented to our plastic surgery team for flap debulking of the right forearm to improve contour (Figures 14, 15).

All case information, including patient demographics, injury, mechanism, preceding orthopedic interventions, flap size, follow-up period, and complications, is summarized in Table 1. Four patients underwent ECS-LDF reconstruction, three of whom were for lower extremity defects and one to treat an upper extremity defect. One case involved a pediatric patient. All injuries resulted from a motor vehicle incident, and the patients sustained severe traumas, including open extremity fractures with overlying large soft tissue defects. All patients underwent fracture fixation with the orthopedic surgery team and ultimately soft tissue reconstruction with the plastic surgery team. Mean hospital stay was 48 days, with a range of 24-63 days.

**Table 1:** Summarized cases information. Patient age (in years), sex, injury, mechanism, preceding orthopedic interventions, flap size, follow-up period, and complications are provided for each case. AvP: auto vs. pedestrian; Fx: fracture(s); LD: latissimus dorsi; LLE: left lower extremity; MVA: motor vehicle accident; NPWT: negative pressure wound therapy; ORIF: open reduction and internal fixation; RLE: right lower extremity; RUE: right upper extremity; M: male; F: female

Case	Patients (age in years and gender)	Injury and mechanism	Orthopedic interventions	ECS-LDF size	Follow-up	Complications and additional operations
1	57 M	Motorcycle; RLE Gustilo IIIB fx, traumatic knee arthrotomy, and soft tissue defect	Debridement, tibia and fibula ORIF, and NPWT	397 cm^2^	2 months	Patient continued to follow-up with the ortho team until he passed away eight months post-operatively; cause of death unknown. Clavien-Dindo classification grade 0 [7].
2	5 F	AvP; left distal femur and proximal tibial fx with degloving	Debridement, femur and tibial ORIF, and NPWT	495 cm^2^	Ongoing	Partial flap necrosis managed with debridement, allograft, and STSG. Hypertrophic scarring at five months post-operative managed with CO_2_ laser x3, steroid injections x3, and tissue expansion and rearrangement. Clavien-Dindo classification grade 3b [7].
3	61 M	AvP; LLE Gustilo IIIB fx with degloving	Debridement, ORIF, and NPWT	160 cm^2^	Limited due to patient transfer back to the admitting hospital post-operatively	None. Unable to classify Clavien-Dindo grade due to lack of sufficient follow-up [7].
4	30 F	MVA; RUE open distal radius and ulnar fx with bony gap, extensor tendon avulsions, nerve injuries, and soft tissue defect	Debridement, tendon and nerve repair, and NPWT	615 cm^2^	Ongoing	None; patient desired flap debulking for improved contour at one year post-operatively. Clavien-Dindo classification grade 0 [7].

The average ECS-LDF size was 416.75 cm^2^ with a standard deviation of 167 cm^2^ among the four patients. One patient’s flap included a vascularized segment of scapular bone measuring 20 cm^2^ (Case 4). A vascular anatomical variation was found in one patient (Case 4), with the thoracodorsal and circumflex scapular arteries both branching off the axillary artery rather than sharing a common source from the subscapular artery.

Primary donor site closure was successful in all four patients. There were no donor site complications, including infection, dehiscence, hematoma, or seroma. There was one complication of partial flap necrosis (Case 2), which was subsequently debrided and repaired with a skin graft.

Two patients required additional operative interventions for improvement of their post-reconstruction healing. One patient developed hypertrophic scarring and therefore benefited from CO_2_ laser therapy, steroid injections, tissue expansion, and local rearrangement. The other patient desired flap debulking to improve the contour of the reconstructed extremity.

## Discussion

Surgical techniques for reconstruction of large upper and lower limb traumatic injuries have been evolving for many years. Historically, amputation was the mainstay of treatment for open fractures with overlying soft tissue loss, but advances in trauma management, skeletal fixation, vascular repair, and microsurgical reconstruction have led to improved outcomes in limb salvage [1,8,9].

Free flaps typically have a higher reported success rate when compared to local flaps in the treatment of more severe limb injuries due to their ability to bring in large amounts of well-vascularized tissue [10,11]. The primary considerations when choosing a free flap are size and the inclusion of necessary tissue components [12,13].

First described in 1982, the scapular fasciocutaneous flap is based on the circumflex scapular artery. It is commonly utilized in extremity reconstruction due to its reliable pedicle and ability to resurface shallow wounds with thin, uniform coverage [14]. The scapular flap can be raised expeditiously, but its main disadvantage is its small size and potential need for position change [15,16]. Thoma and Heddle addressed this limitation with the ECS, which extends the flap beyond the back midline for a maximum skin paddle of up to 39x10 cm [17].

Chimeric flaps consist of various components with separate vascular supplies that ultimately attach to a common pedicle [1]. Surgeons can customize flaps to contain the desired tissues, including muscle, bone, and fasciocutaneous components. The subscapular system provides a variety of options for chimeric flaps. The thoracodorsal and circumflex scapular arteries are branches of the subscapular artery and supply the scapular flap and latissimus dorsi flap, respectively. An ECS can be harvested in combination with LDF to resurface large, composite defects. Because the flaps have independent vascular leashes, the flaps can be inset freely to meet the unique needs of the defect [18].

We utilized a chimeric ECS-LDF to reconstruct four large limb defects with an average size of 416.75 cm^2^. This large size is capable of resurfacing near circumferential limb wounds. To optimize the flap components, we preferentially inset the ECS over anatomic zones that require durable skin and fascia for mobility, such as around the knee joint. The LDF is best utilized to obliterate dead space and cover hardware. Furthermore, bone can be incorporated into the flap design in patients with composite bony defects. We performed one ECS-LDF with vascularized scapular bone supplied by the angular branch of the thoracodorsal artery to reconstruct a 7 cm defect of the radius.

The anatomy of the subscapular system is well described, but there are anatomic variations to be aware of during flap dissection [19]. The subscapular artery typically branches into the circumflex scapular artery and the thoracodorsal artery. However, in 4% of patients, one of our patients had different origins of the thoracodorsal and the circumflex scapular arteries from the axillary artery, which required two anastomoses [6]. In the remaining three patients, each component of the ECS-LDF arose from a single common pedicle, which allowed for a single anastomosis. This is in contrast to other techniques that utilize sequential chimeric perforator flaps to reconstruct massive extremity defects [20]. ECS-LDFs with a single common pedicle enhance operative efficiency and are ideal for use in the vessel-depleted traumatically injured limb.

There were no instances of total flap failure or anastomotic revision; however, one LDF developed partial flap necrosis (Case 2). While the latissimus dorsi muscle typically has a reliable vascular supply, this patient sustained traumatic rib fractures during her initial injury, which likely damaged muscular perforators in the distal flap. The necrotic portion of the flap was debrided and was able to be skin grafted.

Of the three patients who were able to follow-up, two patients (Case 2 and Case 4) achieved a return to normal functioning of the reconstructed limb. Case 1 struggled to establish with physical therapy due to insurance coverage, which complicated his functional recovery. In comparison to other musculocutaneous flaps for lower extremity injuries, such as the anterolateral thigh flap harvested with vastus lateralis, the ECS-LDF is able to achieve limb salvage and restoration of at least some limb function without affecting lower extremity function due to donor site morbidity [17].

The ECS-LDF is unique in its ability to cover extensive soft tissue defects with a single large skin paddle. When designing the ECS skin paddle, we perform a skin pinch to determine the widest flap that can be harvested while achieving direct closure. The flap can also be reliably extended beyond midline and to the anterior axillary line. This allows the freedom to position the pedicle in a central or an eccentric location on the skin paddle, depending on the defect and recipient vessel position. It is our standard practice to perform intra-operative indocyanine green angiography once the ECS is isolated on its pedicle to ensure adequate perfusion to the flap edges. We had no instances of total ECS flap necrosis, and all donor sites were able to be closed primarily with no donor site morbidity.

This contrasts with a series of scapular skin and latissimus dorsi muscle with high documented donor site morbidity [19]. Similarly, Qing et al. reported a series of eight parascapular fasciocutaneous and latissimus dorsi muscle conjoined flaps [20]. However, those with large skin paddles required skin grafting of the donor site [20]. 

Limitations of this case series include a small cohort of four patients and limited long-term follow-up for two of the four patients. Further research of the ECS-LDF should include thorough follow-up regarding return to normal activity and anatomical variations. In the senior author’s opinion, the ECS-LDF allows for coverage of the largest surface area from a single vascular pedicle and donor site while achieving primary closure.

## Conclusions

This case series demonstrates the ability of the ECS-LDF to reconstruct extremity soft tissue defects from traumatic injuries. These flaps are well-suited for reconstructing large, composite limb defects in both adult and pediatric populations due to their robust blood supply and versatile components. This flap design can be customized to include the necessary tissue components while simplifying microvascular anastomosis and minimizing donor site morbidity.
